# An Unusual Presentation of Lyme Neuroborreliosis: A Side-Switching Facial Paralysis in Michigan

**DOI:** 10.51894/001c.165999

**Published:** 2026-07-31

**Authors:** Sneha Rajkumar, Anthony Emmer

**Affiliations:** 1 DMC - Sinai Grace

## 115

### Introduction

Although Lyme disease is becoming more common in the Midwest, it is still not widely recognized as a cause of neurologic symptoms. Facial nerve palsy can occur in Lyme neuroborreliosis; however, it typically presents unilaterally and is accompanied by systemic symptoms or a rash. Sequential or bilateral paralysis is rare and may lead clinicians toward alternative diagnoses. This case describes a young man who developed sequential facial palsy without rash, fever, or a tick bite, illustrating an uncommon presentation of Lyme disease in a non-endemic area.

### Case Presentation

A healthy 23-year-old man presented with left-sided facial weakness, difficulty raising his eyebrow, closing his eye, and smiling. He denied fever, chills, recent travel, or erythema migrans, and he did not recall any tick bites. His only associated symptom was left-sided postauricular and occipital pain. MRI and MRA of the brain were normal, and Lyme IgG/IgM serology was negative. He was diagnosed with Bell’s palsy and started on prednisone and valacyclovir. Three weeks later, he developed right-sided facial weakness that was more severe than the initial left-sided palsy. He remained otherwise neurologically intact with no limb weakness, sensory changes, gait issues, or additional cranial nerve deficits. A lumbar puncture revealed elevated protein levels, mild pleocytosis, and positive intrathecal Lyme antibodies, confirming Lyme neuroborreliosis. He was treated with ocular protection measures, two intravenous doses of ceftriaxone, and a 28-day course of doxycycline. He showed clinical improvement during follow-up.

### Discussion

Sequential bilateral facial palsy is a rare presentation of Lyme disease and accounts for only a small proportion of facial paralysis cases. This pattern of unilateral paralysis followed weeks later by contralateral involvement is particularly uncommon in regions like Michigan, where Lyme disease is not traditionally considered endemic. Early serologic testing may be negative, which can delay diagnosis when the initial presentation resembles idiopathic Bell’s palsy. This case highlights the importance of considering Lyme disease in patients with progressive, persistent, or sequential facial weakness, even in the absence of rash or systemic illness. Growing regional prevalence underscores the need for heightened clinical awareness to ensure timely diagnosis and prevent long-term neurologic complications.

